# Graded pitch profile for the helicoidal broadband reflector and left-handed circularly polarizing cuticle of the scarab beetle *Chrysina chrysargyrea*

**DOI:** 10.1038/s41598-018-24761-w

**Published:** 2018-04-24

**Authors:** A. Mendoza-Galván, L. Fernández del Río, K. Järrendahl, H. Arwin

**Affiliations:** 1 Cinvestav, Unidad Querétaro, Libramiento Norponiente 2000, 76230 Querétaro, Mexico; 2 Department of Physics, Chemistry and Biology, Linköping University, SE-58183 Linköping, Sweden

## Abstract

The cuticle of the beetle *Chrysina chrysargyrea* reflects left-handed polarized light in the broad spectral range from 340 to 1000 nm. Interference oscillations in the experimental Mueller-matrix spectroscopic ellipsometry data reveal that transparent materials comprise the cuticle. A spectral analysis of the interference oscillations makes evident that the pitch profile across the cuticle is graded. The graded pitch and effective refractive indices are determined through non-linear regression analysis of the experimental Mueller matrix by using a cuticle model based on twisted biaxial dielectric slices. Non-uniformity in cuticle thickness as well as in pitch profile near the cuticle surface account for depolarizance of the Mueller matrix. Transmission electron microscopy supports the reliability of the results.

## Introduction

**Figure 1:**
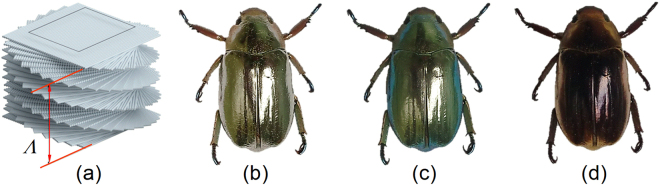
(**a**) Schematics of the Bouligand structure showing the pitch Λ. Photos of the beetle *Chrysina chrysargyrea* taken without polarizer (**b**), with a left-circular polarizer (**c**), and a right-circular polarizer (**d**) in front of the camera.

Metallic sheen in nature stems from the arrangement of nano- or micro-sized structures near the surface of living organisms in the plant and animal kingdoms^1–5^. A special class is the arrangement of nanofibrils forming a helicoidal structure which also is named twisted plywood or Bouligand structure^6^. In this type of structure, the nanofibrils are preferentially oriented in a plane and the orientation of fibrils is slightly twisted between adjacent planes as is shown in Fig. 1(a). The distance after which the planes have completed a twist of 360° defines the pitch (Λ) of the structure. The Bouligand structure has many properties similar to cholesteric liquid crystals. For instance, due to the periodicity the helicoidal structures selectively reflect light at normal incidence in a band centred at wavelength *λ*_0_ = *n*_av_Λ and with a bandwidth Δ*λ* = Δ*n*Λ where *n*_av_ and Δ*n* are the in-plane average refractive index and birefringence, respectively^7,8^. The reflected light is left- or right-handed circularly polarized depending on the handedness of the helicoidal structure. The latter characteristics manifest the circular Bragg phenomenon. Some examples of natural structures showing these properties are the *Pollia condensate* fruit that reflects both left- and right-handed circularly polarized light from cellulose-based helicoidal structures^2,3^ and chitin-based fibrils forming helicoidal structures in some arthropods, particularly in the exoskeleton (cuticle) of many species of beetles^4,5^. So far, only left-handed helicoidal structures have been found in beetles but with specific architectures like in the cuticle of *Chrysina resplendens* right-handed polarized light can also be produced^9,10^.

As was noted above, the in-plane birefringence limits the bandwidth of selective Bragg reflection. However, nature has optimized the cuticle of beetles for multifunctional purposes and broadband reflectors have evolved in several beetles belonging to the *Chrysina* genus^9–27^. Some *Chrysina* beetles look silvery because the Bragg band covers a wide spectral range from the ultraviolet to the near infrared. *Chrysina* beetles with a golden-like appearance can also be found. Both silvery and golden beetles can be found in different specimens of the same species as in *C*. *chrysargyrea*^18^. Figure 1(b) shows a photo of a silvery *C*. *chrysargyrea* specimen taken under illumination with unpolarized light. When a left-handed circular polarizer is put in front of the camera only minor colour changes occur on areas with near-normal incidence as seen in Fig. 1(c). On the sides of the beetle it appears blue and green due to its curvature which is the well-known blue shift at larger angles of incidence. However, a more drastic change is observed when a right-handed circular polarizer is in front of the camera as seen in Fig. 1(d). Instead of having a bright silvery reflection, the cuticle now appears dark brown. These observations qualitatively indicate absence of right-handed circular polarization in the reflected light, thus evidencing the selective Bragg reflection of left-handed polarized light. The weak brown colour is due to scattering of ambient light at large angles of incidence. Broadband reflectors of circularly polarized light have been proposed to originate from a continuous variation of the pitch across the cuticle as imaged by scanning electron microscopy (SEM) and transmission electron microscopy (TEM)^10,15–20^. However, a full explanation of the appealing colours of *Chrysina* beetles and their polarization properties in terms of the pitch structure is challenging. Earlier studies date since more than one hundred years ago^11^ and continued over the last century on *C*. *optima*, *C*. *resplendens* and *C*. *gloriosa*^8–10,12,19^. More recently, this interesting topic has motivated several research groups around the world to perform intensive investigations^13–26^. Some of those studies on beetles of the *Chrysina* genus are based on SEM images and reflectance spectra of unpolarized incident light (*C*. *aurigans*, *C*. *chrysargyrea*)^15–18^. TEM, co-polarized and cross-polarized spectra of right- and left-handed circularly polarized light studies have been reported for *C*. *optima* and *C*. *strasseni*^20^. Mueller-matrix measurements have also been applied to investigate the polarization properties of *Chrysina* beetles (*C*. *chrysargyrea*, *C*. *argenteola*, *C*. *gloriosa*)^21–27^. However, the full capabilities of the Mueller-matrix formalism have not been exploited so far.

Particularly, in a recent work the graded pitch profiles in the cuticle of golden and red *C*. *aurigans* as well as silvery and golden *C*. *chrysargyrea* beetles were determined from SEM images^18^. Using this graded pitch profile, the authors calculated the total reflectance as a sum of two irradiance contributions: one coherent reflection from a perfect left-handed twisted structure and one non-coherent contribution due to variations of the azimuth angle that specifies the orientation of chitin-protein nanofibrils. Fluctuations in the refractive index were also considered in the calculations. In general, the experimental and calculated spectra compare well. However, for silver-like *C*. *chrysargyrea* some discrepancies are noted, mainly at wavelengths shorter than the Bragg reflection. Furthermore, it is not clear how a pitch ranging between 240 and 360 nm produces selective reflection in the near infrared. A complete description of any non-coherent superposition of light beams reflected from the cuticle of beetles requires the use of the Mueller-matrix formalism and advanced modelling to determine the origin of depolarization^28^.

In this work, we determine the graded pitch in the cuticle of silvery *C*. *chrysargyrea* from its depolarizing Mueller matrix. In the analysis, a multilayer model is used to calculate Mueller matrices which then are compared with the experimental matrices. This electromagnetic modelling is based on non-linear regression using the Levenberg-Marquart algorithm as described earlier^29^. The parameters of the model are the thicknesses of the layers comprising the cuticle, the effective refractive indices, and structural parameters describing the graded pitch profile. Non-uniform cuticle thickness and non-uniformity in pitch near the cuticle surface account for depolarization. The complexity of the cuticle requires that the starting values of the cuticle model parameters are sufficiently close to their best-fit values. The overall procedure is therefore to use maxima and minima in interference oscillations in experimental data to extract an approximate pitch profile. Based on its functional dependence on depth, a model pitch profile is then designed and implemented in a helicoidal optical system built with biaxially birefringent slices with step-wise rotation through the cuticle. Refractive index dispersion is then introduced, and regression is performed using the pitch profile parameters determined from interference analysis as starting values. The non-depolarizing model Mueller matrix is finally expanded to include two types of non-uniformities to account for the experimentally observed depolarization. Basics of the Mueller-matrix formalism are given in the Materials and Methods section.

## Results and Discussion

### Overview of experimental Mueller-matrix data

**Figure 2:**
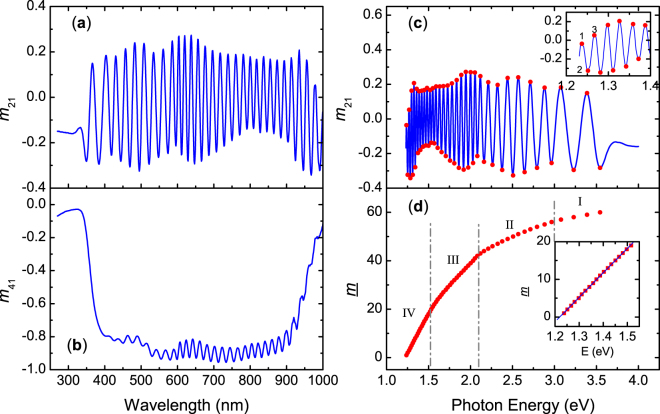
Wavelength dependence of elements (**a**) *m*_21_ and (**b**) *m*_41_ of the Mueller matrix measured at an angle of incidence *θ* = 20° on the elytron of the beetle *C*. *chrysargyrea*. (**c**) *m*_21_ as function of photon energy; the insert in (**c**) shows the labelling of maxima and minima with an index *m*. (**d**) Spectral dependence of *m*; the insert shows a fit to a linear dependence at low photon energies.

The polarization properties of light reflected from the cuticle of the beetle *C*. *chrysargyrea* for unpolarized incident light have been discussed before^26^ but are here briefly recalled. Let us consider incident unpolarized light with Stokes vector [1, 0, 0, 0]^T^ where T means transpose (see equation (8) in Materials and methods). The cuticle of the beetle is represented by its 4 × 4 normalized Mueller matrix (**M**) and the specularly reflected beam is given by the Stokes vector [1, *m*_21_, *m*_31_, *m*_41_]^T^ as obtained from equation (9) in Material and methods. Figure 2 shows elements *m*_21_ and *m*_41_ measured at an angle of incidence of *θ* = 20°. Full Mueller matrices are presented in the Supplementary information. The element *m*_41_ is negative in the wavelength range 340 to 1000 nm which implies reflection of left-handed polarized light for incident unpolarized light. The later result qualitatively explains the silvery appearance of the beetle in Fig. 1(b) and why the beetle looks dark when a right-handed polarizer is placed in front of the camera as seen in Fig. 1(d). The elements *m*_21_ and *m*_31_ are components related to linear polarization, and their oscillatory behaviour is of major importance as will be discussed in the next section. Furthermore, symmetries among the elements of **M**, proper of chiral systems^30^ leave only nine independent elements as is shown in Supplementary Fig. S1. At normal incidence there are further relationships among the nine independent elements that still are fulfilled at low angles of incidence as shown in Supplementary Fig. S2 for *θ* = 20°. Those further relationships show that the spectral information contained in the oscillatory behaviour is equivalent among the elements and *m*_21_ was chosen.

### Effective penetration depth from interference fringes

The clear oscillations observed in the spectra indicate that the electromagnetic waves superpose with enough degree of coherency. That is, light being reflected at different interfaces in the multilayer structure interfere constructively or destructively at specific wavelengths. This interpretation leads us to hypothesize that transparent materials comprise the cuticle of *C*. *chrysargyrea*. If absorption was strong enough, the strength of oscillations would be smeared out or damped. Our first objective is to determine which type of information the interference oscillations contain. For this purpose, we follow the procedure previously applied to data from the cuticle of the beetle *Cotinis mutabilis*^31,32^. Maxima and minima appear in optical spectra of films at discrete wavelengths *λ*_*m*_ where the phase factor equals an integer multiple (*m*) of π, that is,

1 $$\frac{4\pi \,d}{{\lambda }_{m}}\sqrt{{n}_{{\rm{av}}}^{2}-{n}_{{\rm{a}}}^{2}{\sin }^{2}\theta }=m\pi ,$$

where *d* is the thickness of the film and *n*_a_ is the refractive index of the ambient (air). Because the reciprocal relationship between wavelength and interference order in equation (1), a more convenient representation of data is shown in Fig. 2(c) where *m*_21_ is plotted as function of photon energy *E*. According to equation (1), an integer can be associated to each maxima and minima. However, the actual value of *m* has an unknown integer offset. We therefore use a temporary index *m* starting with *m* = 1 at the first maximum observed at the low energy end of the *m*_21_ spectrum as shown in the insert of Fig. 2(c). By determining the energy position of each maxima and minima, the spectral dependence of *m* as shown in Fig. 2(d) is found. A slope decreasing with photon energy is observed and attains an asymptotic behaviour at high photon energies. In Fig. 2(d) we observe changes in the slope of *m* at certain energies which allow identification of four spectral regions denoted I, II, III, and IV to be discussed below. The behaviour of *m* in Fig. 2(d) is different to the one found in data from the cuticle of the beetle *C*. *mutabilis*^31,32^ which only showed discrete pitch regions.

The negative values of *m*_41_ seen in Fig. 2(b) shows that *C*. *chrysargyrea* reflects left-handed polarized light at *θ* = 20° in the spectral range where interference oscillations appear in *m*_21_. These oscillations indicate that there is a fictitious resonance cavity corresponding to the penetration depth of the left-handed polarization. This is an attribute of a circular Bragg reflector which we will refer to as an attenuation length in the discussion below. Therefore, *d* in equation (1) can be interpreted as an effective penetration depth $\langle \eta \rangle$ and we set *d* = <*η*>. Refractive indices of transparent materials are real-valued and normally show wavelength dispersion. However, for simplicity we neglect any dispersion in *n*_av_ in the preliminary analysis but will introduce it later. With this assumption and by using *E*_*m*_ = 1240/*λ*_*m*_ for *E*_*m*_ in eV and *λ*_*m*_ in nm, a linear relationship between *m* and *E*_*m*_ is found from equation (1). By taking the photon energy derivative of *m* from equation (1) we obtain,

2 $$\langle \eta \rangle =\frac{1240}{4\sqrt{{n}_{{\rm{av}}}^{2}-{n}_{{\rm{a}}}^{2}{\sin }^{2}\theta }}\frac{d\underline{m}}{d{E}_{m}},$$

where $\langle \eta \rangle$ is given in nm. The insert in Fig. 2(d) shows a linear fit (d*m*/d*E*_*m*_ constant) at low photon energies (region IV) and considering *n*_av_ = 1.6 as typical values for *Chrysina* beetles^9^, we obtain $\langle \eta \rangle$ = 12.9 μm from equation (2). The objective below is to refine the analysis and determine the spectral variation of $\langle \eta \rangle$.

### Spectral location of the minimum penetration depth and in-depth variation of pitch

**Figure 3:**
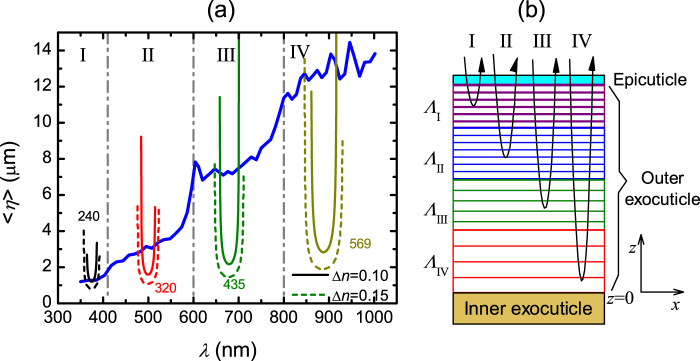
(**a**) Wavelength dependence of the effective penetration depth <*η*> as calculated from equation (2); four spectral regions I–IV are identified. The U-shaped curves are the calculated attenuation length *η* for selected pitch values between 240 and 569 nm and two values of in-plane birefringence (Δ*n*). (**b**) Schematic representation of the cuticle of the beetle *C*. *chrysargyrea* deduced from the spectral dependence of <*η*>.

Figure 3 shows $\langle \eta \rangle$ versus *λ*_*m*_ in more detail and for the full spectral range as obtained from equation (2) by numerically taking the photon energy derivative of *m* in Fig. 2(d). A general increase with wavelength is observed but step-like changes in $\langle \eta \rangle$ can be identified at certain wavelengths and are marked with vertical dash-dot lines separating the four spectral regions denoted I, II, III, and IV introduced already in Fig. 2(d). On the other hand, for a left-handed Bouligand structure it is known that in a band of selective Bragg reflection, the wave vector component ${K}_{||}^{{\rm{LH}}}$ parallel to the helix axis of the selectively reflected left-handed mode becomes complex-valued and its imaginary part defines the characteristic field attenuation length according to $\eta =1/\text{Im}\{{K}_{||}^{{\rm{LH}}}\}$ with the in-depth variation of the field proportional to *e*^−ζ/*η*^ where ζ is the distance from the surface^7^. The wave vector ${K}_{||}^{{\rm{RH}}}$ for the right-handed mode is however real-valued and propagation of the right-handed component occurs without attenuation. Outside a band of selective Bragg reflection both right- and left-handed modes propagate without attenuation. In Fig. 3, the U-shaped curves correspond to *η* calculated for selected pitch values with *n*_av_ = 1.6 and two values of Δ*n*. These calculations were performed at *θ* = 20° within the two-wave approximation for light propagation in a semi-infinite chiral media at oblique incidence^33–35^. Previously, we have applied this approach to analyse data measured on the cuticle of the scarab beetle *C*. *mutabilis*^31,32^. In Fig. 3 it can be noticed that *η*_min_, the minimum value of *η*, increases with the pitch and that a larger in-plane birefringence broadens the selective Bragg reflection and decreases *η*_min_.

Clearly, in Fig. 3 the smaller values of $\langle \eta \rangle$ are found in region I and are coincident with *η*_min_ for Λ_I_ = 240 nm and Δ*n* = 0.1. In region II both, left- and right-handed modes, propagate without attenuation through the Bouligand structure with pitch Λ_I_ near the cuticle surface but the left-handed mode is selectively reflected from helicoidal structures with a continuously increasing pitch. Because region II is centred at wavelength 500 nm, the average pitch is Λ_II_ = 320 nm as determined from ${\lambda }_{m}={n}_{{\rm{av}}}{\rm{\Lambda }}\,\cos \,{\theta }_{{\rm{t}}}$, where *θ*_t_ is the angle of wave propagation inside the helicoidal structure determined from Snell’s law ${n}_{{\rm{a}}}\,\sin \,\theta ={n}_{{\rm{av}}}\,\sin \,{\theta }_{{\rm{t}}}$^36^. Passing from region II to region III <*η*> steeply increases and electromagnetic waves of wavelength between 600 and 800 nm travel without attenuation about 7 μm through the cuticle. However, in region III selective reflection is produced by Bouligand structures with average pitch Λ_III_ = 435 nm as calculated from *λ*_III_ = 680 nm. The largest penetration depth is found in region IV centred at *λ*_IV_ = 890 nm where selective reflection comes from helicoidal structures with average pitch Λ_IV_ = 569 nm located more than 10 μm deep in the cuticle. The cuticle model in Fig. 3 is a schematic representation of the increasing pitch with depth in the cuticle deduced in this paragraph from the analysis of <*η*> versus *λ*_*m*_. In summary, light with wavelength 350 to 400 nm only penetrates roughly 1 μm at λ = 375 nm into the exocuticle. In the spectral region 400 to 600 nm, the top 2–3 μm of the cuticle is transparent, but the left-handed mode is then selectively reflected with a penetration depth of roughly 1.5 μm into region II at λ = 500 nm (the U-formed curve in Fig. 3). Similar phenomena occur in regions III and IV.

### Mathematical representation of the graded helicoidal pitch profile

**Figure 4:**
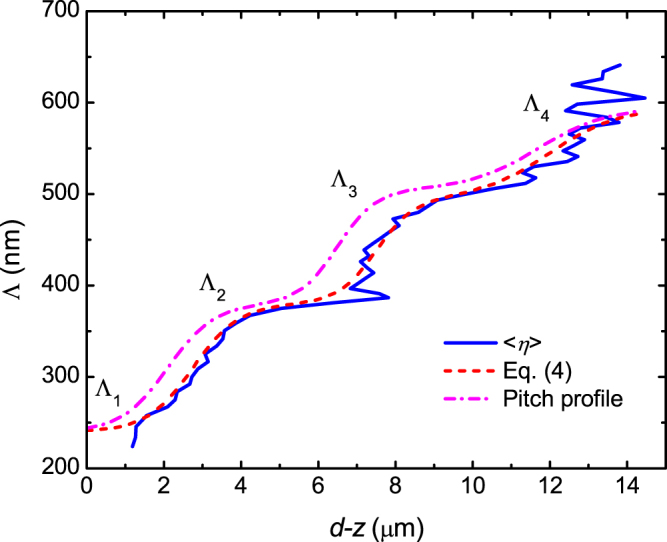
Pitch Λ versus outer exocuticle depth *d-z* determined form the effective penetration depth <*η*> in Fig. 3 (solid line). The dashed line shows Λ calculated from equation (4) with the parameters in Supplementary Table S1 whereas an assumed pitch profile calculated from equation (4) with parameters in parenthesis in Supplementary Table S1 is given by the dash-dot line. See the text for details.

Further insight into the pitch profile in the cuticle is given in Fig. 4 which is Fig. 3 redrawn with the vertical and horizontal axes interchanged and rescaled. In Fig. 4 the horizontal axis is renamed to $d-z=\langle \eta \rangle$ where *d* is the outer exocuticle thickness and *z* is the distance from the bottom of the outer exocuticle as defined in Fig. 3. The wavelength axis in Fig. 3 is transformed to the Λ-axis in Fig. 4 according to

3 $${\rm{\Lambda }}={\lambda }_{m}/({n}_{{\rm{av}}}\,\cos \,{\theta }_{{\rm{t}}}).$$

In this way Fig. 4 illustrates pitch versus cuticle depth *d*-*z*. As can be noticed, Λ shows an increasing step-like dependence with depth which can be described with a logistic function given by,

4 $${\rm{\Lambda }}(d-z)={{\rm{\Lambda }}}_{1}+{\sum }_{j=2}^{4}\frac{{\rm{\Delta }}{{\rm{\Lambda }}}_{j}}{1+{e}^{-(\frac{d-z-{\eta }_{0j}}{{\gamma }_{j}})}},$$

where ΔΛ_*j*_, *η*_*0j*_, and γ_*j*_ are, respectively, the strength, centre, and broadening of the steps between pitch Λ_*j*-1_ and Λ_*j*_. The graded pitch (dashed line) in Fig. 4 was calculated with equation (4) and parameters values as given in Supplementary Table S1. These values were obtained by trial and error, but they could also be obtained by fitting if more accuracy is needed. Three depth zones with nearly constant values of the pitch (Λ_1_, Λ_2_ and Λ_3_) are identified in Fig. 4 and a fourth zone is estimated (Λ_4_). In each of these zones the orientation of nanofibrils comprising the Bouligand structure changes at a constant ratio (2π/Λ_*j*_) with depth as illustrated in Fig. 1(a). On the other hand, in between the cuticle zones where Λ continuously increases (e.g. at the transition ${{\rm{\Lambda }}}_{1}\to {{\rm{\Lambda }}}_{2}$), the orientation of nanofibrils changes in a decreasing ratio with depth. Apparently, the pitch profile in the cuticle of *C*. *chrysargyrea* is described by Fig. 4. However, as the interference observed in data of Fig. 2 does not originate from physical cavities but is due to penetration lengths of the left-handed mode, we need to consider effects of light propagation in samples of finite thickness and the finite bandwidth of selective Bragg reflection. By doing this, it is possible to determine the actual location of the steps between pitches.

Consider a zone where Λ_*j*_ is nearly constant extending in the cuticle over a length *L*_*j*_ and characterized by the corresponding *η*_min, *j*_. According to data in Figs 3 and 4 we have *L*_*j*_ ∼ *η*_min, *j*_. Thus, incident light of wavelength in the band of selective Bragg reflection due to Λ_*j*_ probes not only the full length *L*_*j*_, but also deeper zones of slightly larger pitch because the finite bandwidth of selective reflection. On the other hand, the continuous change of pitch in zones at the transition ${{\rm{\Lambda }}}_{1}\to {{\rm{\Lambda }}}_{2}$, in a first approximation can be linear with depth. The selective Bragg reflection of a chiral structure with a linear variation of the pitch as a function of sample thickness has been investigated by other authors^37^. In that work, numerical calculations were performed at normal incidence for a structure with an in-plane birefringence of Δ*n* = 0.225 which is larger than what is used here. In the present work, consider a value of Λ at the transition ${{\rm{\Lambda }}}_{1}\to {{\rm{\Lambda }}}_{2}$, located at cuticle depth *d*-*z*_03_. The left-handed mode of incident light of wavelength fulfilling the condition $\lambda ={n}_{{\rm{av}}}{\rm{\Lambda }}\,\cos \,{\theta }_{{\rm{t}}}$ will penetrate further a distance *l* where the pitch has increased to the value Λ + ΔΛ. The value of *l* is limited by the overlap of bandwidths of selective Bragg reflection due to Λ and Λ + ΔΛ. For the first and second steps, we assume *l* to have a value corresponding to about twice the value of Λ because a sample of two periods in thickness is enough to produce (a broad and weak) selective Bragg reflection in a single period chiral structure of in-plane birefringence Δ*n* = 0.13 as reported by other authors^38^. However, the third step at 12 μm was shifted by a smaller quantity because it is close to the estimated outer exocuticle thickness of 13.5 μm which leaves two to three times the pitch at this depth where Λ_IV_ = 569 nm. Therefore, the assumed pitch profile was shifted as shown with the dash-dot line in Fig. 4 with the parameters given in parenthesis in Supplementary Table S1. In summary, the analysis of spectral positions of maxima and minima in the spectrum of the Mueller-matrix element *m*_21_ has provided evidence for the pitch profile across the cuticle of *C*. *chrysargyrea*. In the next section, we use this result to perform a fit to the experimental Mueller matrix to determine structural parameters and optical functions in the cuticle more precisely.

### Electromagnetic modelling of the graded pitch profile in the cuticle of *C*. *chrysargyrea*

Modelling the Bouligand structure is accomplished by subdividing the cuticle in biaxial slices with equal thickness and with a step-wise rotation around the *z*-axis of the laboratory coordinate frame defined in Fig. 3. Each slice is characterized with effective refractive indices (*n*_1_, *n*_2_, *n*_3_) referred to the principal axes, i.e. *n*_1_ and *n*_2_ are the in-plane refractive indices and *n*_3_ is the index in the *z*-direction. Referring to the Bouligand structure of Fig. 1(a), *n*_1_ and *n*_2_ are located parallel and perpendicular to the fibrils and *n*_3_ along the helical axis. The direction of *n*_1_-axis defines an orientation angle *ϕ* (in degrees) with respect to the *x*-axis and has a value *ϕ*_0_ at *z* = 0. We assign a graded *z*-variation in $\phi (z)$ as described below. The cumulated number of periods, i.e. full 360° turns, is given by ${N}_{{\rm{p}}}(z)=(\phi (z)-{\phi }_{0})/360^\circ$. In the special case of a linear variation of *ϕ* with *z*, the pitch is given by Λ = *z*/*N*_p_(*z*), i.e. the inverse of the (constant) slope of *N*_p_(*z*). Generally, we have variation in pitch with *z* given by

5 $${\rm{\Lambda }}(z)={(\frac{d{N}_{{\rm{p}}}}{dz})}^{-1}.$$

For electromagnetic modelling of the cuticle structure we consider a variation in *ϕ*(*z*) such that equation (5) produces a sigmoid-like variation with *z* at the transitions in pitch in accordance with the qualitative analysis in the previous section. We use

6 $$\phi (z)={\phi }_{0}+360\frac{T}{d}[z+{\sum }_{j}{a}_{j}\,\mathrm{ln}(1+{e}^{\frac{z-{z}_{0j}}{{b}_{j}}})],$$

where *T* is the number of turns for the single-pitch case, *d* is the cuticle thickness, *a*_*j*_, *z*_0*j*_, and *b*_*j*_ have, respectively, equivalent meaning to ΔΛ_*j*_, *η*_*0j*_, and γ_*j*_ in equation (4). The initial values of the parameters *a*_*j*_, *b*_*j*_, and *z*_0*j*_, were those reproducing the dash-dot pitch profile in Fig. 4 and determined as described in Supplementary Fig. S3. First, the layer thickness was set to *d* = 13.5 μm which is approximately the limit value of $\langle \eta \rangle$ in Fig. 3. From the values *η*_*0j*_ given in parenthesis in Supplementary Table S1 we get ${z}_{0j}=(d-{\eta }_{0j})$. Setting *a*_*j*_ = 0 in equation (6) leads to ${N}_{{\rm{p}}}=zT/d$ and the number of turns calculated according to equations (5) and (6) becomes $T=d/{{\rm{\Lambda }}}_{{\rm{IV}}}$ = 23.7 if we use Λ_IV_ = 0.569 μm as deduced from Fig. 3. So far, we have considered a constant value of *n*_av_. However, variation with wavelength should be introduced for physically meaningful refractive indices. As explained in the Materials and Methods section, refractive indices with Cauchy dispersion were considered. Values of refractive index and pitch profile parameters are given in Supplementary Table S2.

### Determining the cuticle structure of *C*. *chrysargyrea* by regression analysis with non-uniform exocuticle thickness

The model described in the previous section accounts for a non-depolarizing system. However, Mueller matrices measured on the cuticle of beetles show depolarization. Indeed, attempts to fitting the experimental data with the ideal model were unsuccessful manifested as to large amplitude on the oscillations. This is evidencing the importance of considering deviations from an ideal system. Because non-coherent superposition of light reflected from different parts of the cuticle with different thickness produce depolarization, we consider non-uniformity in cuticle thickness. This type of non-ideal feature in the analysis is implemented in the commercial software. Forward calculations show that the major effect of a non-uniform thickness is a damping of the amplitude of the oscillations. A non-uniformity in thickness of 1.7% was found to produce oscillations with amplitude similar to those in the experimental data. Leaving the non-uniformity in thickness as a fitting parameter, the fitting was statistically equivalent. The regression analysis was performed in the same fit procedure using data from measurements at *θ* = 20 and 50°. Data from larger angles of incidence probe a larger area and the effects of non-uniformity are stronger and are not included. The starting value of the epicuticle thickness was *d*_epi_ = 350 nm corresponding to values reported for other *Chrysina* beetles^20^, whereas the starting value of *ϕ*_0_ was chosen to match the oscillations in the spectrum of *m*_21_. More details on the regression procedure are given in the Materials and Methods section. The experimental and best-fit Mueller matrices are in very good agreement as is shown in Supplementary Fig. S4.

**Figure 5:**
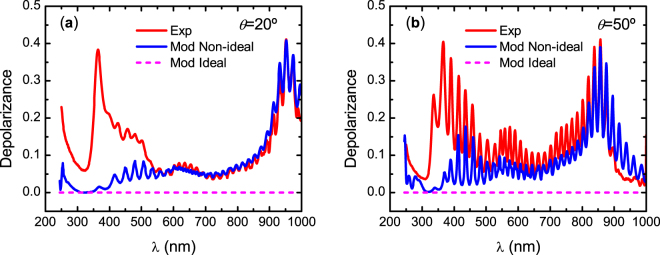
Experimental and model-calculated depolarizance of Mueller matrices at angles of incidence (**a**) *θ* = 20° and (**b**) *θ* = 50° assuming a non-uniformity in thickness of 1.7%. An ideal model is non-depolarizing as indicated by the dashed line.

To investigate how well the non-uniformity in thickness accounts for the deviation from an ideal non-depolarizing Mueller matrix, we calculated the depolarizance *D* of **M**, which is an average measure of the depolarization produced by a system for all incident pure states and is given by^39^,

7 $$D=1-{P}_{{\rm{\Delta }}}=1-{[\frac{1}{3}(\frac{tr({{\bf{M}}}^{{\rm{T}}}{\bf{M}})}{{M}_{11}^{2}}-1)]}^{1/2}$$

where *P*_Δ_ is the degree of polarimetric purity (also called depolarization index), T stands for transpose, and *tr* stands for trace. Figure 5 shows the depolarizance of the experimental and modelled Mueller matrices at *θ* = 20 and 50°. First, we observe that with a uniform thickness the depolarization is zero. For reference Mueller matrices for such ideal system are shown in Supplementary Fig. S5. However, by implementing a non-uniformity in thickness, the difference between the experimental and model-calculated depolarizance for λ > 550 nm is considerably reduced. However, a thickness non-uniformity does not explain the depolarizance of the measured **M** at shorter wavelengths, which corresponds to the same spectral range where the experimental and fitted data differ in amplitude as shown in the Supplementary Fig. S4. Below we investigate the source of depolarization at shorter wavelengths.

### Non-uniformity in pitch near the surface of the cuticle of *C*. *chrysargyrea*

**Figure 6:**
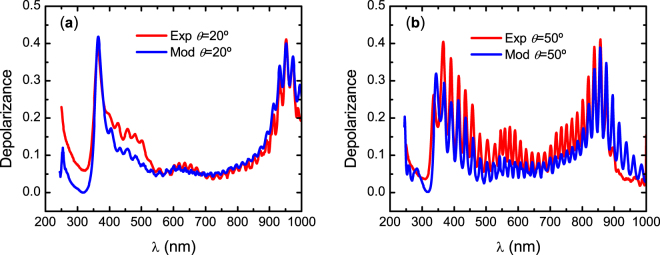
Experimental and model-calculated depolarizance of Mueller matrices at angles of incidence (**a**) *θ* = 20° and (**b**) *θ* = 50° assuming a non-uniformity in exocuticle thickness as well as in pitch distribution (smearing) near the cuticle surface.

To identify the source of depolarization in the wavelength range 245–550 nm, we first notice that this spectral range overlaps with regions I and II in Fig. 3 where the penetration depth is about 4 μm. Therefore, an additional non-ideal feature in the chiral structure producing depolarization below wavelength 550 nm should be introduced. This was accomplished by smearing the position of the step accounting for pitch change at *z*_03_. Smearing of *z*_03_ means that the parameter is varied within values in a width Δ*z*_03_. This variation introduces non-uniformity in pitch near the cuticle surface. Thus, the non-coherent superposition of light reflected from regions with different pitch values produces depolarization. The parameters of the epicuticle and anisotropic slabs were fixed to those determined before. We comment here that smearing of pitch was previously successfully applied in modelling the cuticle structure of the beetle *C*. *aurata*^29^. The best fit was obtained with Δ*z*_03_ = 0.25 μm. As shown in the Supplementary Fig. S6, the description of the data in **M** below 550 nm was improved. The improvement of the model is confirmed by calculating the depolarizance. As seen in Fig. 6, the non-uniformity in pitch near the cuticle surface gives an excellent description of the experimental data.

### Graded pitch profile in beetle cuticle of *C*. *chrysargyrea*

**Figure 7:**
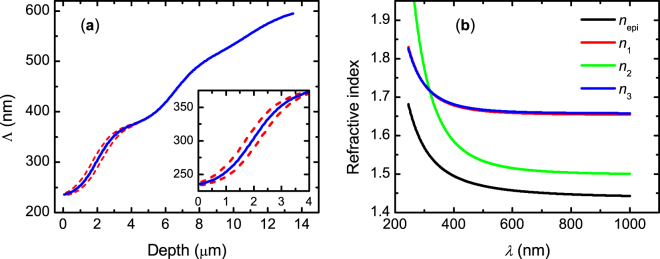
(**a**) Pitch profile across the outer exocuticle of *C*. *chrysargyrea* determined from the regression analysis of Mueller-matrix data. (**b**) Refractive indices of epicuticle (*n*_epi_) and principal components (*n*_1_, *n*_2_, *n*_3_) of the anisotropic slices modelling the outer exocuticle. The insert in (**a**) shows the pitch variation near the cuticle surface accounting for depolarization at wavelength shorter than 550 nm.

The optimized depth profile of pitch found by regression analysis is shown in Fig. 7(a). The epicuticle and outer exocuticle thicknesses are 361 nm and 13.6 μm, respectively, and the number of turns *T* = 23.6. Values of all fitted parameters and confidence limits are given in Supplementary Table S3 and the correlation matrix is shown in Supplementary Table S4. More than 90% of the correlation values are |*c*_*ij*_| < 0.6. As can be noticed in Fig. 7(a), the pitch ranges between 250 and 600 nm. This variation is in accordance with that reported for the beetle *C*. *strasseni* determined with TEM^20^. It is here also relevant to quote Neville, “…from a constructional viewpoint a broad band reflector demands less accuracy of layer spacing control than does a narrow bandwidth reflector…”^12^. Other authors reported a graded pitch ranging between 240 and 360 nm from the analysis of SEM images taken on the cuticle of silver-like *C*. *chrysargyrea*^18^. This variation of pitch is not consistent with the reflection of left-handed polarized light in the near infrared. For example, taking the largest pitch (360 nm) and 1.6 as an estimate of the refractive index used by the authors in ref.^18^. the wavelength of Bragg reflection is 576 nm.

The change of pitch in the near surface region is shown in the insert in Fig. 7(a) with dashed lines. This type of non-uniformity was observed by other authors in TEM images of the cuticle of *C*. *strasseni*^20^. Quoting MacDonald *et al*., “…in *C*. *strasseni* the exocuticle extends to a depth of approximately 15.5 μm, and generally comprises 40 helicoidal pitches, although sometimes an additional helicoid or two are observed as the structure accounts for the curvature of the elytron…”^20^. Here, we have arrived at a similar result by modelling the cuticle as a non-ideal system and quantified the depolarizance of the Mueller matrix of *C*. *chrysargyrea*. This is a unique capability of the Stokes-Mueller formalism.

### Optical functions of beetle cuticle

The effective refractive indices determined from the regression analysis are shown in Fig. 7(b). They are in the range of previously reported data from other beetles^9,29^. At λ = 1000 nm the in-plane birefringence is maximum ${\rm{\Delta }}n={n}_{1}-{n}_{2}=0.16$ and decreases for shorter wavelengths becoming zero at 320 nm. Some authors associate the relative large birefringence to the presence of uric acid^9,18^. In ref.^18^, the in-plane effective birefringence is determined as ${\rm{\Delta }}n=(1-f){\rm{\Delta }}{n}_{{\rm{c}}}+f{\rm{\Delta }}{n}_{{\rm{ua}}}$ where Δ*n*_ua_ = 0.31 and Δ*n*_c_ = 0.04 are the birefringence values of uric acid and chitin, respectively, and *f* = 0.59 is the volume fraction of uric acid. However, X-ray diffraction data and infrared spectroscopy as shown in Supplementary Figs S7 and S8, respectively, do not show presence of uric acid but further investigation is in progress.

### Transmission electron microscopy

**Figure 8:**
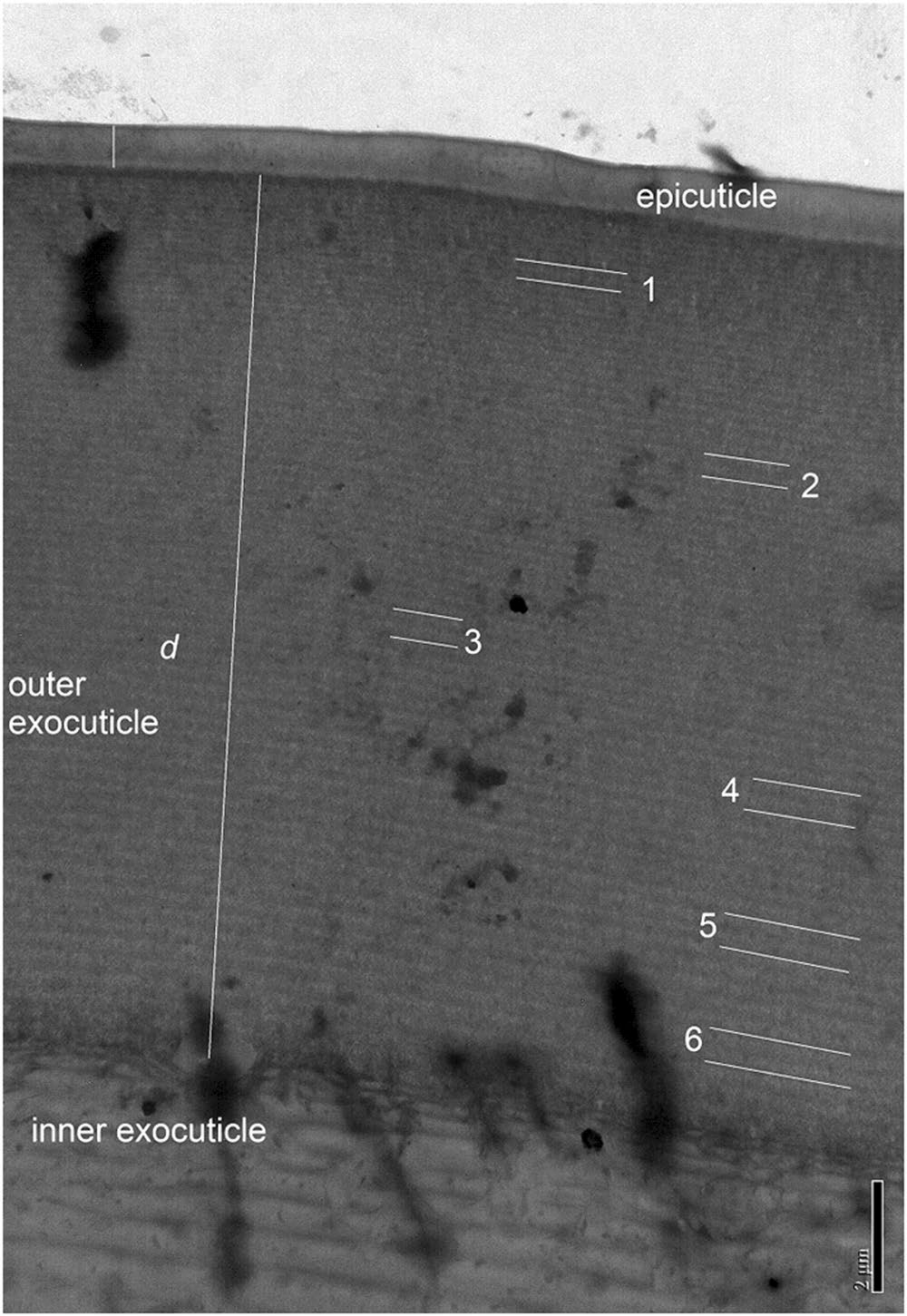
TEM image of a cuticle cross section of *C*. *chrysargyrea* showing the epicuticle, the outer exocuticle and part of the inner exocuticle. The scale bar is 2 µm and the numbers refers to positions were the pitch was evaluated (see text).

TEM was performed to corroborate the results obtained from the regression analysis of Mueller-matrix data. Figure 8 shows a TEM image taken on the cuticle of a specimen of *C*. *chrysargyrea*. For the specimen analysed, the epicuticle and outer exocuticle thicknesses are approximately 740 nm and 15.8 µm, respectively. Although the size of the area of the cuticle imaged (≈8 µm) is smaller than the dimension of the probe light beam (≈100 µm), non-uniformity in thickness is observed which is in agreement with the model accounting for depolarization. Clearly, a multilayer structure is found in the outer exocuticle as revealed by dark and light lamellae. In the image, the pitch corresponds to the distance between two dark (bright) lamellae. In Fig. 8, at positions 1 to 6 at depths of about 1.1, 4.1, 7.5, 9.8, 12.3, and 14.3 µm, the pitch was evaluated and found to be 318, 416, 516, 577, 600, and 605 nm, respectively. In summary, the regression analysis performed provides reliable details on the graded pitch profile in beetle cuticle.

We have determined microstructural parameters and optical functions producing the polarization and depolarization properties of the cuticle of *C*. *chrysargyrea*. For optical biomimetism appropriate selection of materials and processing is required to obtain broadband reflectors^40^. The results of this work could serve as a guide to reproduce the outstanding optical performance of the cuticle of *C*. *chrysargyrea*. Furthermore, this could inspire the development of applications for security issues by encoding depolarization through precise control of the structure during materials processing.

## Conclusions

The cuticle of the beetle *C*. *chrysargyrea* is a broadband reflector of left-handed polarized light. Interference oscillations in Mueller-matrix data contain information about the graded pitch profile across the cuticle. The pitch monotonically increases from the surface towards the inner exocuticle. The depolarizing experimental Mueller matrix was reproduced by modelling the cuticle as a stack of biaxial slabs with a step-wise rotation of the azimuth angle of the in-plane principal refractive indices and with non-uniformity of cuticle thickness. An additional non-uniformity in pitch in the near-surface region accounts for a second source of depolarization in the Bragg reflection at short wavelengths.

## Materials and Methods

### Mueller-matrix measurements

Mueller-matrix measurements were performed using a dual rotating compensator ellipsometer (RC2, J. A. Woollam Co., Inc.) that allows determination of the 15 elements of a normalized Mueller matrix. Focusing probes were used reducing the size of the beam to less than 100 µm. More details on the instrument are found in^23–27,29^. The specular measurements were carried out at angles of incidence (*θ*) (measured from the normal to the surface) from 20 to 75° in steps of 5° in the wavelength (*λ*) range of 245 to 1000 nm. The acquisition time was 30 s at each *θ*. The measurements were done on the elytra, which are the wing covers, of the scarab beetle *Chrysina chrysargyrea* (Salle, 1874) which is found in Costa Rica and Panama. The specimen studied was collected in Monteverdi, Costa Rica, in 2011 and kindly provided by Dr. Parrish Brady (University of Texas at Austin). Crab chitin and uric acid (Sigma Aldrich) were used as received.

### Basics of Stokes-Mueller formalism

This formalism provides a complete description of the polarization and depolarization properties of light-matter interaction^28,41^. Light beams are described by Stokes vectors with components,

8 $${\bf{S}}=[\begin{array}{c}I\\ Q\\ U\\ V\end{array}],$$

where *I* = *I*_p_ + *I*_s_ accounts for the total irradiance. *Q* = *I*_p_ − *I*_s_ and *U* = *I*_+45°_ − *I*_−45°_ are irradiances describing linear polarization. Here, p is parallel to and s is perpendicular to the plane of incidence and + 45° and −45° are measured from the plane of incidence. The fourth component *V* = *I*_R_ − *I*_L_ accounts for circular polarization where R and L stands for right- and left-handed, respectively. It holds that $I\ge \sqrt{{Q}^{2}+{U}^{2}+{V}^{2}}$ where the equal sign is valid for a completely polarized beam whereas the other case is valid for a partially polarized beam. The polarization and depolarization capabilities of a sample are contained in its 4 × 4 Mueller matrix (**M**) with elements *M*_*ij*_ (i,j = 1..4). The Stokes vectors of the incident (**S**_*i*_) and reflected (**S**_*r*_) light beams are related by^28,41^,

9 $${{\bf{S}}}_{r}={\bf{M}}{{\bf{S}}}_{i}=[\begin{array}{cccc}{M}_{11} & {M}_{12} & {M}_{13} & {M}_{14}\\ {M}_{21} & {M}_{22} & {M}_{23} & {M}_{24}\\ {M}_{31} & {M}_{32} & {M}_{33} & {M}_{34}\\ {M}_{41} & {M}_{42} & {M}_{43} & {M}_{44}\end{array}]{{\bf{S}}}_{i}.$$

Mueller matrices from samples reflecting completely polarized beams for any incident completely polarized beam are non-depolarizing (ideal). In contradistinction, if the reflected light is partially polarized for some incident polarization states, the Mueller matrix is depolarizing. In general, depolarization arises from the incoherent superposition of light beams. In this work, we use Mueller matrices normalized to *M*_11_ (*m*_*ij*_ = *M*_*ij*_/*M*_11_) and incident Stokes vectors with *I* = 1.

### Cuticle refractive indices

Because electronic transitions in the UV range and molecular vibrations in the mid IR range are outside of the spectral range of measurements, only normal dispersion is expected. In particular, the effects of IR resonances can be neglected. The three indices *n*_1_, *n*_2_, and *n*_3_ of the biaxial outer exocuticle may therefore be modelled with Cauchy expressions,

10 $${n}_{\alpha }={A}_{\alpha }+{B}_{\alpha }/{\lambda }^{2}+{C}_{\alpha }/{\lambda }^{4}\,(\alpha =1,2,3),$$

where *A*_*α*_, *B*_*α*_ and *C*_*α*_ are fitting parameters and λ is expressed in μm. As a good initial choice, it is required that *n*_1_ = *n*_3_>*n*_2_ to account properly for the sign of *m*_31_ which is noticed mostly at large angles of incidence. The epicuticle is assumed isotropic with refractive index ${n}_{{\rm{epi}}}={A}_{{\rm{epi}}}+{B}_{{\rm{epi}}}/{\lambda }^{2}+{C}_{{\rm{epi}}}/{\lambda }^{4}$ and for the inner exocuticle we used the same refractive index dispersion as was done for the beetle *Cetonia aurata*^29^. Initially we set the same value for all *B* and *C* parameters with the exemption of *C*_2_ which was set larger to account for a decreasing birefringence at shorter wavelengths. To avoid unphysical refractive indices, all Cauchy parameters were defined to be positive.

### Electromagnetic modelling

In summary, the set of model parameters for the regression analysis is {**X**} = {*T*, *ϕ*_0_, *d*, *d*_epi_, *A*_epi_, *B*_epi_, *C*_epi_, *A*_*α*_, *B*_*α*_, *C*_*α*_, *a*_*j*_, *b*_*j*_, and *z*_0*j*_}, i.e. parameters in equations (6) and (10) as well as epicuticle thickness (*d*_epi_) and Cauchy coefficients of the refractive index *n*_epi_. With such large number of fitting parameters, one would assume that correlation effects become severe. However, the data base is large with full Mueller matrices recorded at multiple angles in a wide spectral range. Furthermore, the dispersion parameters are spectrally global, and the pitch parameters are independent among each transition as they are spectrally local. Optimization of model parameters was carried out in three steps. (i) We can only within certain limits guess values on the model parameters {*ϕ*_0_, *d*_epi_, *A*_epi_, *B*_epi_, *C*_epi_, *A*_*α*_, *B*_*α*_, *C*_*α*_} and this sub-set was fitted first using the values of parameters in the sub-set {*T*, *d*, *a*_*j*_, *b*_*j*_, *z*_0*j*_} which were estimated from experimental data. (ii) A fitting was then performed on the sub-set {*T*, *ϕ*_0_, *d*, *a*_*j*_, *b*_*j*_, *z*_0*j*_} keeping the values fixed of the parameters optimized in step (i). (iii) Finally, the full set {**X**} was optimized. It should be mentioned that in step (i) we obtained *B*_1_ = *B*_3_ = 0 and these parameters were not further varied in step (iii) where it was also found that *B*_2_ = 0. Non-linear regression where performed by fitting parameters in the model representing the sample to minimize the difference between experimental and model-generated Mueller-matrix data using the Levenberg-Marquardt algorithm as described in more detail elsewhere^29^. The regression was performed with the CompleteEASE software (J. A. Woollam Co., Inc.).

### Complementary characterization

X-ray diffraction data were acquired with a Rigaku/Dmax2100 equipment equipped with Cu radiation (K_α1_ = 1.5406 Å). Infrared spectra were measured with a Spectrum GX system/Perkin Elmer using a resolution of 4 cm^−1^ and averaging over 64 scans. Spectra from the cuticle of *C*. *Chrysargyrea* and crab chitin were measured in attenuated total reflection mode and for uric acid the KBr pellet technique was employed. For transmission electron microscopy the sample was first fixed in 3% glutaraldehyde for 2 h, then rinsed in sodium cacodylate buffer, immersed in osmium tetroxide for 1 h, rinsed in deionized water, immersed in 1% uranyl acetate for 1 h, rinsed again in deionized water, followed by a dehydration series starting with 30% ethanol and ending with 100% ethanol, and embedding in a resin. The sample was imaged in JEOL 100 S TEM instrument.

## Electronic supplementary material

Supplementary Information
