# Supplementary material for: Graded pitch profile for the helicoidal broadband reflector and left-handed circularly polarizing cuticle of the scarab beetle Chrysina chrysargyrea
Source: Sci Rep. 2018 Apr 24;8:6456. doi: 10.1038/s41598-018-24761-w (PMC5915428; doi:10.1038/s41598-018-24761-w)
Supplement: Supplementary file 1 — Supplementary Information [file 41598_2018_24761_MOESM1_ESM.doc]

Supplementary Information

Graded pitch profile for the helicoidal broadband reflector and left-handed circularly polarizing cuticle of the scarab beetle *Chrysina* *chrysargyrea*

A. Mendoza-Galván^1^, L. Fernández del Río^2^, K. Järrendahl^2^, H. Arwin^2^

*^1^Cinvestav, Unidad Querétaro, Libramiento Norponiente 2000, 76230 Querétaro, Mexico*

*^2^Department of Physics, Chemistry and Biology, Linköping University, SE-58183 Linköping, Sweden*

**Contents**

**Supplementary figures:**

**Figure S1.** Symmetries among the elements of the Mueller matrix of *C. chrysargyrea.*

**Figure S2.** Phase relationships among the elements of the Mueller matrix of *C. chrysargyrea.*

**Figure S3.** Broadening the selective Bragg reflection band with a graded pitch profile.

**Figure S4.** Experimental and best-fit Mueller matrices assuming a non-uniformity in thickness.

**Figure S5.** Experimental and best-fit Mueller matrices assuming a uniform thickness.

**Figure S6.** Experimental and best-fit Mueller matrices assuming a non-uniformity in exocuticle thickness as well as a pitch distribution (smearing) near the cuticle surface.

**Figure S7.** X-ray data diffraction data from the cuticle of the beetle *C*. *chrysargyrea* and of uric acid (C_5_H_4_N_4_O_3_).

**Figure S8.** Infrared absorbance spectra from the cuticle of the beetle *C*. *chrysargyrea*, uric acid (C_5_H_4_N_4_O_3_) and crab chitin.

**Supplementary tables:**

**Table S1**. Parameters used in equation (4) generating the graded pitch profile (dashed line) in Fig. 4.

**Table S2**. Refractive index and pitch profile parameters employed to generate data in Fig. S3.

**Table S3.** Values and confidence limits of fitted parameters.

**Table S4**. Correlation matrix of fitting parameters.

**References**

**Figure S1**. Symmetries among the elements of the Mueller matrix measured on the cuticle of *C. chrysargyrea* at *θ*=20º. It is seen that *m*_12_=*m*_21_, *m*_13_=-*m*_31_, *m*_14_=*m*_41_, *m*_23_=-*m*_32_, *m*_24_=*m*_42_, and *m*_34_=‑*m*_43_ which corresponds to that of a chiral system [1]. Remains thus only nine symmetry-independent elements in **M**.

**Figure S2**. Phase relationships of oscillations among the elements of **M** measured on the cuticle of *C. chrysargyrea* at *θ*=20º. We observe a constant difference in phase between *m*_12_ and *m*_31_; *m*_31_, *m*_41_, *m*_22_, *m*_23_, and *m*_43_ are in phase; *m*_33_ and *m*_44_ are out of phase by π radians with respect to *m*_31_; *m*_24_ is out of phase by π radians with respect to *m*_21_. In summary, all contain the same phase information. The data were vertically displaced by arbitrary values only to highlight the phase relationships. The vertical dashed lines are drawn as a guide to the eye.

**Table S1**. Parameters used in equation (4) generating the graded pitch profile (dashed line) in Fig. 4. The constant term is Λ_1_=240 nm. Values in parenthesis are used to generate the dash-dotted profile in Fig. 4.

| Step | *η_0j_* (μm) | ΔΛ*_j_* (nm) | γ*_j_* (μm) |
| --- | --- | --- | --- |
| 1 | 2.75 (2.10) | 140 (140) | 0.6 (0.6) |
| 2 | 7.5 (6.5) | 115 (125) | 0.5 (0.5) |
| 3 | 12.0 (11.7) | 100 (90) | 0.9 (0.9) |

**Figure S3**. (a) Illustration of the stepwise broadening of the Bragg reflection band due to changes in pitch at different cuticle depths. (b) Graded pitch profile determined from the rotation of the *n*_1_-axis according to equations (5) and (6). The discrete values marked Λ*_j_* (j=1,2,3) correspond to those in Fig. 4. Forward calculations using *a_j_*=0, *d*=13.5 μm, *T*=23.7 in equation (6) and *φ*_0_=0 produce the selective reflection band at long wavelengths as shown in panel (a) by the red curve and marked Λ_4_. The refractive indices used in the calculations were represented with Cauchy dispersion functions as described in the Materials and Methods section. In the next step, *a*_1_ and *b*_1_ in equation (6) were determined by constraining the jump in the pitch to give $\Lambda_{4}\to\Lambda_{3}$, as shown in panel (b). This leads to a further broadening of the Bragg reflection as shown in panel (a) by the orange curve. The remaining parameters in equation (6), i.e. *a*_2_, *b*_2_, *a*_3_, and *b*_3_ were obtained in a similar way and full values are shown in Table S1. The broadening of the Bragg reflection in each step can be observed in panel (a) and the corresponding pitch profile is shown in panel (b). It should be noticed that if the pitch distribution is calculated from equation (5) by inserting equation (6) in the expression for *N*_p_, a functional dependence of the graded pitch (as proposed in Fig. 4) similar to that in equation (4) is obtained in the vicinity of each transition.

**Table S2**. Refractive index and pitch profile parameters employed to generate data in Fig. S3.

| Epicuticle | *A*_epi_ | *B*_epi_ | *C*_epi_ |
| --- | --- | --- | --- |
| *n*_epi_ | 1.45 | 0.001 | 0.001 |
|  |  |  |  |
| Exocuticle | *A_α_* | *B_α_* | *C_α_* |
| *n*_1_ | 1.67 | 0.001 | 0.0001 |
| *n*_2_ | 1.53 | 0.001 | 0.001 |
| *n*_3_ | 1.67 | 0.001 | 0.0001 |
|  |  |  |  |
| Pitch profile | *a*_j_ (μm) | *z*_0j_ (μm) | *b*_j_ (μm) |
| *j*=1 | 0.14 | 1.76 | 0.81 |
| *j*=2 | 0.22 | 7.02 | 0.54 |
| *j*=3 | 0.46 | 11.7 | 0.46 |

**Figure S4**. Experimental (red solid lines) and best-fit (blue dashed lines) Mueller matrices at *θ*=20º (a) and *θ*=50º (b) using a non-uniformity in thickness of 1.7%. The scale is shown in the lower-left panel. Notice that the fitted and experimental data are in a very good agreement, particularly regarding the phase of the oscillations. Differences in amplitude are still observed, however. Model parameters and confidence limits are given in Table S2.

**Figure S5**. Experimental (red solid lines) and model calculated (blue dashed lines) Mueller matrices at *θ*=20º (a) and *θ*=50º (b) assuming a uniform thickness. Model parameters are given in Table S2. The scale is shown in the lower-left panel.

**Figure S6**. Experimental (red solid lines) and best-fit (blue dashed lines) Mueller matrices at *θ*=20º (a) and *θ*=50º (b) assuming a non-uniformity in both, exocuticle thickness and pitch distribution (smearing) near the cuticle surface. The scale is shown in the lower-left panel. Notice that the discrepancy observed in the amplitude of oscillations in some calculated elements below 550 nm of Fig. S4 practically disappears.

**Table S3.** Values and confidence limits of fitted parameters.

| *d* (μm) | *d*_epi_ (nm) | *φ*_0_ (deg) | *T* |
| --- | --- | --- | --- |
| 13.622 ± 0.008 | 361± 1 | 121± 2 | 23.3 ± 0.1 |
|  |  |  |  |
| Refractive indices |  |  |  |
| Epicuticle | *A*_epi_ | *B*_epi_ | *C*_epi_ |
| *n*_epi_ | 1.436 ± 0.007 | 0.006 ± 0.001 | 0.0005 ± 1E-04 |
|  |  |  |  |
| Exocuticle | *A_α_* | *B_α_* | *C_α_* |
| *n*_1_ | 1.654 ± 0.001 | 0 | 0.00063 ± 1E-05 |
| *n*_2_ | 1.498 ± 0.002 | 0 | 0.00222 ± 3E-05 |
| *n*_3_ | 1.657 ± 0.001 | 0 | 0.00061 ± 2E-05 |
|  |  |  |  |
| Pitch profile | *a*_j_ (μm) | *z*_0j_ (μm) | *b*_j_ (μm) |
| *j*=1 | 0.273 ± 0.020 | 2.90 ± 0.13 | 1.29 ± 0.04 |
| *j*=2 | 0.345 ± 0.025 | 7.35 ± 0.03 | 0.82 ± 0.03 |
| *j*=3 | 0.538 ± 0.018 | 11.83 ± 0.01 | 0.58 ± 0.01 |

**Table S4**. Correlation matrix of fitting parameters.

|  | *d*_epi_ | *A*_epi_ | *B*_epi_ | *C*_epi_ | *A*_1_ | *C*_1_ | *A*_2_ | *C*_2_ | *A*_3_ | *C*_3_ | *φ*_0_ | *T* | *a*_1_ | *z*_01_ | *b*_1_ | *a*_2_ | *z*_02_ | *b*_2_ | *a*_3_ | *z*_03_ | *b*_3_ | *d* |
| --- | --- | --- | --- | --- | --- | --- | --- | --- | --- | --- | --- | --- | --- | --- | --- | --- | --- | --- | --- | --- | --- | --- |
| *d*_epi_ | 1.000 | -0.207 | -0.064 | -0.073 | 0.009 | 0.091 | 0.073 | -0.090 | 0.150 | 0.124 | 0.020 | -0.056 | -0.019 | -0.051 | -0.004 | -0.014 | -0.076 | -0.027 | 0.278 | 0.447 | 0.270 | -0.180 |
| *A*_epi_ | -0.207 | 1.000 | -0.892 | 0.844 | 0.089 | 0.067 | -0.033 | -0.011 | 0.047 | 0.005 | -0.013 | 0.035 | 0.113 | 0.156 | 0.134 | -0.105 | -0.070 | -0.095 | -0.094 | -0.353 | -0.104 | -0.195 |
| *B*_epi_ | -0.064 | -0.892 | 1.000 | -0.966 | -0.128 | -0.091 | 0.025 | 0.098 | -0.119 | -0.040 | -0.004 | -0.034 | -0.106 | -0.146 | -0.121 | 0.127 | 0.089 | 0.121 | 0.004 | 0.323 | -0.001 | 0.257 |
| *C*_epi_ | -0.073 | 0.844 | -0.966 | 1.000 | 0.133 | 0.058 | -0.033 | -0.091 | 0.105 | 0.011 | 0.007 | 0.048 | 0.094 | 0.140 | 0.101 | -0.115 | -0.067 | -0.108 | -0.053 | -0.401 | -0.039 | -0.221 |
| *A*_1_ | 0.009 | 0.089 | -0.128 | 0.133 | 1.000 | -0.311 | 0.134 | 0.043 | 0.771 | 0.034 | 0.065 | 0.216 | -0.061 | 0.087 | -0.059 | 0.037 | 0.100 | 0.051 | -0.090 | -0.013 | -0.066 | -0.754 |
| *C*_1_ | 0.091 | 0.067 | -0.091 | 0.058 | -0.311 | 1.000 | 0.109 | -0.107 | -0.031 | -0.105 | 0.020 | -0.142 | 0.068 | -0.017 | 0.052 | -0.071 | -0.045 | -0.084 | 0.101 | 0.045 | 0.072 | 0.029 |
| *A*_2_ | 0.073 | -0.033 | 0.025 | -0.033 | 0.134 | 0.109 | 1.000 | 0.713 | 0.566 | -0.100 | -0.171 | -0.455 | -0.027 | -0.318 | -0.073 | 0.046 | -0.258 | 0.007 | 0.101 | -0.076 | 0.093 | -0.596 |
| *C*_2_ | -0.090 | -0.011 | 0.098 | -0.091 | 0.043 | -0.107 | 0.713 | 1.000 | 0.203 | 0.021 | -0.026 | -0.272 | -0.124 | -0.291 | -0.151 | 0.143 | -0.188 | 0.115 | -0.078 | -0.104 | -0.076 | -0.308 |
| *A*_3_ | 0.150 | 0.047 | -0.119 | 0.105 | 0.771 | -0.031 | 0.566 | 0.203 | 1.000 | -0.166 | -0.063 | -0.079 | -0.017 | -0.066 | -0.031 | 0.007 | -0.057 | -0.004 | 0.047 | -0.004 | 0.055 | -0.870 |
| *C*_3_ | 0.124 | 0.005 | -0.040 | 0.011 | 0.034 | -0.105 | -0.100 | 0.021 | -0.166 | 1.000 | 0.067 | 0.043 | 0.003 | 0.034 | 0.009 | -0.007 | 0.023 | -0.002 | 0.040 | 0.106 | 0.037 | -0.001 |
| *φ*_0_ | 0.020 | -0.013 | -0.004 | 0.007 | 0.065 | 0.020 | -0.171 | -0.026 | -0.063 | 0.067 | 1.000 | 0.505 | -0.455 | 0.004 | -0.516 | 0.401 | 0.224 | 0.449 | -0.390 | 0.133 | -0.342 | -0.155 |
| *T* | -0.056 | 0.035 | -0.034 | 0.048 | 0.216 | -0.142 | -0.455 | -0.272 | -0.079 | 0.043 | 0.505 | 1.000 | -0.262 | 0.455 | -0.281 | 0.197 | 0.526 | 0.295 | -0.449 | 0.087 | -0.376 | 0.115 |
| *a*_1_ | -0.019 | 0.113 | -0.106 | 0.094 | -0.061 | 0.068 | -0.027 | -0.124 | -0.017 | 0.003 | -0.455 | -0.262 | 1.000 | 0.725 | 0.959 | -0.969 | 0.269 | -0.959 | 0.526 | -0.222 | 0.477 | 0.001 |
| *z*_01_ | -0.051 | 0.156 | -0.146 | 0.140 | 0.087 | -0.017 | -0.318 | -0.291 | -0.066 | 0.034 | 0.004 | 0.455 | 0.725 | 1.000 | 0.639 | -0.743 | 0.654 | -0.660 | 0.146 | -0.142 | 0.152 | 0.062 |
| *b*_1_ | -0.004 | 0.134 | -0.121 | 0.101 | -0.059 | 0.052 | -0.073 | -0.151 | -0.031 | 0.009 | -0.516 | -0.281 | 0.959 | 0.639 | 1.000 | -0.909 | 0.100 | -0.902 | 0.566 | -0.195 | 0.517 | 0.012 |
| *A*_2_ | -0.014 | -0.105 | 0.127 | -0.115 | 0.037 | -0.071 | 0.046 | 0.143 | 0.007 | -0.007 | 0.401 | 0.197 | -0.969 | -0.743 | -0.909 | 1.000 | -0.232 | 0.990 | -0.577 | 0.228 | -0.539 | 0.010 |
| *z*_02_ | -0.076 | -0.070 | 0.089 | -0.067 | 0.100 | -0.045 | -0.258 | -0.188 | -0.057 | 0.023 | 0.224 | 0.526 | 0.269 | 0.654 | 0.100 | -0.232 | 1.000 | -0.181 | -0.401 | -0.006 | -0.393 | 0.099 |
| *b*_2_ | -0.027 | -0.095 | 0.121 | -0.108 | 0.051 | -0.084 | 0.007 | 0.115 | -0.004 | -0.002 | 0.449 | 0.295 | -0.959 | -0.660 | -0.902 | 0.990 | -0.181 | 1.000 | -0.576 | 0.244 | -0.528 | 0.019 |
| *A*_3_ | 0.278 | -0.094 | 0.004 | -0.053 | -0.090 | 0.101 | 0.101 | -0.078 | 0.047 | 0.040 | -0.390 | -0.449 | 0.526 | 0.146 | 0.566 | -0.577 | -0.401 | -0.576 | 1.000 | 0.335 | 0.991 | -0.066 |
| *z*_03_ | 0.447 | -0.353 | 0.323 | -0.401 | -0.013 | 0.045 | -0.076 | -0.104 | -0.004 | 0.106 | 0.133 | 0.087 | -0.222 | -0.142 | -0.195 | 0.228 | -0.006 | 0.244 | 0.335 | 1.000 | 0.338 | 0.020 |
| *b*_3_ | 0.270 | -0.104 | -0.001 | -0.039 | -0.066 | 0.072 | 0.093 | -0.076 | 0.055 | 0.037 | -0.342 | -0.376 | 0.477 | 0.152 | 0.517 | -0.539 | -0.393 | -0.528 | 0.991 | 0.338 | 1.000 | -0.072 |
| *d* | -0.180 | -0.195 | 0.257 | -0.221 | -0.754 | 0.029 | -0.596 | -0.308 | -0.870 | -0.001 | -0.155 | 0.115 | 0.001 | 0.062 | 0.012 | 0.010 | 0.099 | 0.019 | -0.066 | 0.020 | -0.072 | 1.000 |

**Figure S7.** X-ray data diffraction data from (a) the cuticle of the beetle *C*. *Chrysargyrea* and (b) uric acid (C_5_H_4_N_4_O_3_). Labelling of diffraction lines in (a) correspond to α-chitin [2]. In (a) the sharp peak at 34.8º (*) is due to the sample support. In (b) the diffraction lines correspond to the monoclinic structure of uric acid lattice parameters *a*=14.464 Å, *b*=7.403 Å, *c*=6.208 Å, and *β*=65.10° [3].

**Figure S8.** Infrared absorbance spectra measured on the cuticle of the beetle *C*. *chrysargyrea* from the epicuticle and endocuticle sides. For clarity, the spectra are split in two spectral ranges (a) and (b). In (b) characteristic amide bands (I, II, and III) of proteins are identified [4]. For comparison, (c) and (d) show the spectra of crab chitin and uric acid C_5_H_4_N_4_O_3_. Absorption bands for uric acid agree with reference data [5]. In (d) amide bands I and II are characteristic of chitin and the split of amide I in two bands identifies α-chitin [2].

**References**

1. Arteaga, O. Natural optical activity vs circular Bragg reflection studied by Mueller matrix ellipsometry, *Thin Solid Films* **617**, 14-19 (2016).
2. Minke, R. & Blackwell, J. The Structure of α-chitin, *J*. *Mol*. *Biol*. **120**, 167-181 (1978).
3. Ringertz, H. The molecular and crystal structure of uric acid, *Acta Cryst*. **20**, 397-403 (1966).
4. Iconomidou, V. A., Chryssikos, G. D., Gionis, V. Willis, J. H. & Hamodrakas, S. J. “Soft”-cuticle protein secondary structure as revealed by FT-Raman, ATR FT-IR and CD spectroscopy, *Insect Biochem*. *Mol*. *Biol*. **31**, 877-885 (2001).
5. <http://webbook.nist.gov/cgi/cbook.cgi?Scan=cob9737&Type=IR>
